# Developing Countries Must Invest in Access to Information for Health Improvements

**DOI:** 10.2196/jmir.5.1.e5

**Published:** 2003-03-31

**Authors:** Akintola B Odutola

**Affiliations:** ^1^Centre for Health Policy & Strategic StudiesLagosNigeria

That many developing countries, especially in sub-Saharan Africa, have low health status is not news. What is of interest and germane to the process of public health development and reform in these countries is sustainable ideas and proposals for health improvements. Among the many proposals discussed in international health circles, access to appropriate and credible information is topical. Why is this so?

Systematically-harnessed information and communication technologies have been shown to improve the health of populations in many developed countries through empowerment of those who access and use information — from the simple homemaker and working mother to the highest-ranking policymaker. These technologies have also been shown to democratize the public space by fostering freedom of choice and expression as well as rapid access to and sharing of information. These highly-cherished values of participatory democracy have in turn clearly helped to engender better health in individuals and communities. Fascinating and beneficial as these technologies may sound, the deep-rooted factors militating against access to information and the daunting challenges thereto in developing countries are well known [1,2]. Developing countries have generally not invested in access to information as much as they should, even within their available means. Indeed, they are being left behind (the so-called digital gap or digital divide). Very little benefit is accruing to developing countries from the incredible and bountiful digital world. But these countries need to benefit for many good reasons. For example, Ade Lucas, Adjunct Professor of Public Health at Harvard School of Public Health, recently addressed the daunting health challenges existing in most developing countries today. He demonstrated clearly how available evidence-based information — when accessed and used appropriately in the public-policy domain — can make a difference to policy choices and decision-making in the development and implementation of HIV/AIDS prevention programs in developing countries [3]. Regrettably, for many reasons that are often recounted but not always tenable, most developing-country governments and people have not harnessed available information and communication technologies to improve public health in their respective countries. In volunteering a solution, Lucas asked a rhetorical question: "How can African governments tackle their daunting health problems especially in the face of limited financial resources?" His answer: "they must adopt health policies that are based on good information and must use the most [evidence-based] and cost-effective interventions in their programmes" [3].

There is no question that good and evidence-based information is available worldwide and that the Internet is providing both the tools and platform for low-cost, area-wide, and effective dissemination and retrieval of such information. Availability of information is one thing, access to and use of the available information is another. Behind Lucas' advocacy is a clear call for a rethink of the public-health policy-making process in most developing countries where it is not uncommon to misplace priorities and place emphasis on tertiary care and high technology procurement to the detriment of primary health care — including appropriate information provision.

For developing countries to achieve the benefits of access to health information, they must invest strategically in information production, gathering, storage, dissemination, and public health literacy promotion. In most developing countries, where the total per capita spending on health is less than $15 per year [4], is this an economically viable investment? The answer must be a resounding "Yes" bearing in mind the multiplicative empowering impact of appropriate information on millions of people, compared to the few hundreds or thousands that white-elephant tertiary hospitals may benefit per time frame.

How may the rethink for investment in access to health information be achieved in developing countries? Herein lies a challenging role for development and multilateral institutions and agencies like the World Health Organization (WHO) and other United Nations (UN) organizations, as well as the various research and leadership systems in developing countries. Given the global impact of UN multilateral institutions in development matters, a call for them to enunciate policies and foster programs of investment towards access to information in developing countries would not be out of place.

Similarly, just as access to development aid, loans, and grants is now being linked by governments and institutions in the North to "good governance" in developing countries, these same institutions and their developing-nation counterparts should commit to fostering investment in information infrastructure in developing countries as a prerequisite for health and development grants, loans, and aids.

In these nonexclusive ways, the developing world may begin to tackle the daunting and challenging problems of promoting cost-effective and efficient health improvements through improved information access among other ways and means.
